# Patients’ expectations of coming home with Very Early Supported Discharge and home rehabilitation after stroke - an interview study

**DOI:** 10.1186/s12883-015-0492-0

**Published:** 2015-11-16

**Authors:** Åsa Nordin, Katharina S. Sunnerhagen, Åsa B. Axelsson

**Affiliations:** Institute of Neuroscience and Physiology, Sahlgrenska Academy, University of Gothenburg, Gothenburg, Sweden; Institute of Health and Care Sciences, Sahlgrenska Academy, University of Gothenburg, Gothenburg, Sweden; University of Gothenburg Centre for Person-Centred Care (GPCC), Sahlgrenska Academy, University of Gothenburg, Gothenburg, Sweden; Sunnaas Rehabilitation Hospital, Nesodden, Oslo Norway

**Keywords:** Coming home, Content analyses, Early supported discharge, Home rehabilitation, Interviews, Patients’ expectations, Qualitative, Stroke, Very early supported discharge

## Abstract

**Background:**

An Early Supported Discharge (ESD) and rehabilitation from a coordinated team in the home environment is recommended in several high-income countries for patients with mild to moderate symptoms after stroke. Returning home from the hospital takes place very early in Sweden today (12 days post stroke), thus the term Very Early Supported Discharge (VESD) is used in the current study. The aim of this study was to describe patients’ expectations of coming home very early after stroke with support and rehabilitations at home.

**Method:**

This is an interview study nested within a randomized controlled trial; Gothenburg Very Early Supported Discharge (GOTVED), comparing VESD containing a home rehabilitation intervention from a coordinated team to conventional care after stroke. Ten participants (median age 69) with mild to moderate stroke symptoms (NHISS 0 to 8 points) were recruited from the intervention group in GOTVED. Interviews were conducted 0–5 days before discharge and the material was analyzed with qualitative content analysis.

**Results:**

Four main categories containing 11 subcategories were found. The VESD team was expected to provide *“Support towards independency”*, by helping the participants to manage and feel safe at home as well as to regain earlier abilities. The very early discharge gave rise to expectations of coming home to *“A new and unknown situation”,* causing worries not to manage at home and to leave the safe environment at the ward. A fear to suffer a recurrent stroke when being out of reach of immediate professional help was also pronounced. In contrast to these feelings of insecurity and fear, “*Returning to one’s own setting”* described the participants longing home, where they would become autonomous and capable people again. They expected this to facilitate recovery and rehabilitation. *“A new everyday life”* waited for the participants at home and this was expected to be challenging. Different strategies to deal with these challenges were described.

**Conclusions:**

The participants described mixed expectations such as insecurity and fear, and on the other hand, longing to come home. Moreover, they had a high degree of confidence in the expected support of the VESD team. The health professionals at the hospital may build on this trust to reduce the patients’ insecurity for coming home. In addition, it may be beneficial to explore the patients’ expectations thoroughly in front of discharge, as certain feelings and thoughts could complicate or support the home coming process. Thus, a greater attention on such expectations may facilitate the patient’s transition from hospital to home after stroke.

## Background

Approximately 16 million people suffer a stroke worldwide each year [1]. It is one of the major causes of disability adjusted life years (DALY’s) lost in high-income countries [2], affecting motor, cognitive and emotional functions as well as having an overall impact on the existential dimension of the person [3].

In Sweden, most patients suffering a stroke are being discharged home after the hospital stay [4]. In front of discharge, patients have reported feeling inadequately prepared to return home [5]. Discharge and the first few months at home is a time of transition and patients report great uncertainty about the future during this period [5, 6]. Coming home brings the person face to face with the consequences of the stroke, as their functional limitations becomes concrete [7]. This has been described as a struggle because of the changes in body functions and therefore their engagement in daily activities, as well as the emotional impact that these changes have on the individual [5].

Early Supported Discharge (ESD) and follow-up rehabilitation at home is shown to be beneficial for a selected group of patients after stroke, i.e. those with minor to moderate stroke symptoms [8, 9]. For these patients, ESD accelerates discharge from the hospital, resulting in a shorter hospital stay, while the support is offered within the community setting from either a hospital or community based coordinated team who are experienced in stroke rehabilitation [8, 9]. Many high-income countries, including Sweden, recommend the provision of ESD service [10–13].

Long term dependency in the Activities of Daily Living (ADL) has been found to be reduced with ESD, with the greatest benefits for those with less severe disability [8, 9]. There are, however, conflicting findings with regards to patient satisfaction with an ESD, with some studies reporting improved satisfaction with an ESD [8, 9], while another conclude that there is insufficient evidence for this improved satisfaction [14]. The initial mean hospital stay before discharge, for patients receiving an ESD was found to vary from 9.8 to 42 days, according to a recent literature review [14]. The average hospital stay after stroke in Sweden today is 12 days and 55 % of the patients are thereafter discharged directly to their homes [4]. Accordingly, many patients in Sweden are being discharged home very early after stroke and we are therefore using the term very early supported discharge (VESD) in the current study.

In previous studies, ESD has been examined predominantly in order to investigate effectiveness and costs compared to conventional care [8, 9, 15] However, there are three studies focusing specifically on patients’ experiences in the context of ESD [16–18]. Of those, two studies described the patients’ experiences of the home coming and the home rehabilitation. According to these studies, coming home early after stroke was perceived positively by the majority of the patients, while the home-based rehabilitation was described as both being positive and having areas in need of improvement. Interviews were performed addressing the patients’ experiences, on average 70 days [16] and 6 to 8 months after discharge [17]. However, the patients’ expectations of coming home very early after stroke with support and rehabilitation at home have not yet been described.

A greater knowledge of the patients’ expectations, thoughts and ideas about coming home very early after stroke with a supported discharge and rehabilitation at home is needed. This knowledge may facilitate home coming, make the first period less uncertain and improve the rehabilitation process.

The aim of this study was to describe patients’ expectations of coming home very early after stroke with support and rehabilitations at home.

## Methods

This is an interview study nested within a randomized controlled trial; GOTVED [19], comparing VESD containing a therapeutic rehabilitation intervention from a coordinated multidisciplinary team with conventional care after stroke. The study was approved by the Regional Ethics Committee of the Western region of Sweden and informed written consent was obtained from all the participants.

### Participants

The participants were recruited from the intervention group in the GOTVED study [19]. The inclusion criteria of the GOTVED study were: confirmed stroke according to WHO’s criteria [20], ≥18 years of age, living within 30 min drive from the stroke unit, and mild to moderate remaining stroke symptoms at day 2; i.e. National Institute of Health Stroke Scale (NIHSS) [21] score 0–16, Barthel Index BI [22] score 50–99. If BI score was maximal = 100, Montreal Cognitive Assessment (MoCA) [23] should be <26.

Exclusion criteria were life expectancy <1 year and an inability to speak or communicate in Swedish before the stroke event. Fourteen consecutive participants in GOTVED were approached and informed about this nested interview study by an occupational therapist who was not involved in this part. All 14 agreed to be interviewed. Of those, three could not participate; two due to logistical reasons and one who was discharged to a nursing home.

During the interviews, one participant did not want to answer the questions and was excluded from the study. Therefore, the population in this study consisted of 10 participants. Nine of the participants had suffered a stroke for the first time, while one participant had had a prior stroke 10 years ago with no residual symptoms. One participant had severe aphasia (2 points on item 9, NIHSS), while three participants had mild-to-moderate or severe dysarthria (1–2 points on item 10, NHISS). Participant characteristics are summarized in Table 1.

**Table 1:** Demographic and clinical characteristics of the participants (*n* = 10)

Characteristics	
Female/male, n	4/6
Age, median (range)	69 (63–95)
Living alone/sharing household, n	5/5
Housing, apartment/house, n	6/4
Time at stroke unit (days), median (range)	12 (5–17)
Ischemic/hemorrhagic stroke, n	10/0
Stroke Severity, NIHSS (0–42):^a^	
Moderate stroke (5–15), n	2
Minor stroke (1–4), n	5
No stroke symptoms, n	3
Barthel Index (0–100), median (range)^b^	87.5 (50–100)
MoCa (0–30), median (range)^b^	22 (15–27)

^a^At arrival to hospital. ^b^At inclusion to study (day 2)

*NIHSS* National Institute of Health Stroke Scale, *MoCa* Montreal cognitive assessment

### Intervention

The VESD began at the stroke unit with a meeting to make a plan for coming home. The stroke nurse coordinating the VESD team, other involved health professionals, the patient and sometimes the next of kin participated. At this meeting, the patients’ needs and wishes were explored and their personal goals with rehabilitation were formulated and decided. After discharge from the stroke unit, the intervention comprised 2–4 VESD team visits (stroke nurse, physiotherapist, occupational therapist) per week, for a maximum of four weeks. The patient and the VESD team decided the time to end the intervention within this time frame. For further information about the intervention, see the GOTVED study protocol [19].

### Data collection

The first author (ÅN), who is a physiotherapist with > 4 years of clinical experience of stroke rehabilitation, gathered the interview data from February 2012 to March 2014. This was done under the guidance of the last author (ÅBA), who is a nurse and associate professor with > 15 years of experience of doing qualitative research. There was no relationship between the interviewer and the participants prior to the study. A single interview was performed 0–5 days before discharge from the stroke unit. It took place in a secluded room at the stroke unit. With regards to the participants with aphasia and dysarthria, the interviews were adapted in order to support communication. The adaption was based on the interviewer’s knowledge and experience of working with this group of patients in clinical settings. The interviews lasted around 30 min (average 28 min, range 9 to 53 min), were audio recorded and transcribed verbatim. Three of the interviews were noticeably shorter than the others (<15 min). The data material showed both similarities and variations in expectations, though fulfilling the criteria for performing a qualitative content analysis.

### Interview guide

An interview guide with open-ended questions was used, covering the following two areas:

1. Expectations of coming home
  What thoughts do you have about coming home?
  How do you think it will work at home?
2. Expectations of the VESD
  What thoughts do you have about the support and training you will receive at home?
  What are your wishes with the rehabilitation service you will receive?

Follow-up questions were asked specific to the participants’ comments in order to enrich and give a better understanding of the answers.

### Data analysis

A conventional qualitative content analysis inspired by Krippendorf [24] and Hsieh and Shannon [25] was performed by the first author (ÅN). The analysis was mainly descriptive, aiming to systematically organize the data into a structured format. The text material was analyzed in the following five steps:

1. The interviews were read repeatedly to get a sense of the content in its entirety.
2. Meaning units (text segments that convey interesting information in relation to the research question) were derived from the text, condensed and labelled with a code capturing the key concept of the text.
3. The codes were abstracted and sorted into subcategories and categories based on how the different codes were related.
4. The interviews were read again to check for the adequacy of the categories and subcategories with regards to the content of the interviews.
5. Citations for each subcategory were identified from the data.

As an example, the meaning unit”It will be fine for me because I’ll be getting help at home (from the VESD team)” was labelled “Help at home” and sorted in to the subcategory “Getting support to manage at home”. Thereafter, this subcategory was sorted in to the category “Support towards independency”. The analysis process moved back and forth between the different stages and between the whole interview and parts of the text to see that categories, subcategories, codes and citations made sense. The last author (ÅBA) reviewed the interview transcripts and checked the relevance of the codes, the subcategories and categories. Discrepancies between the authors were discussed until a consensus agreement was reached. The computer software programme NVivo 10 (QSR International) was used to organize the data.

## Results

**Fig. 1:**
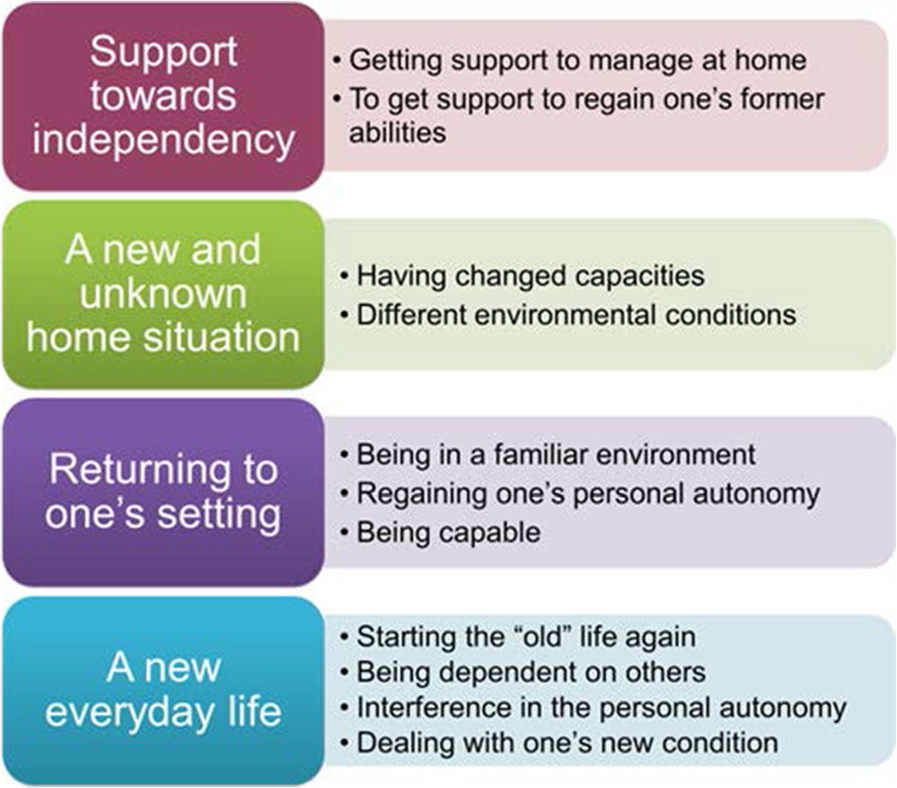
Patients’ expectations of coming home with Very Early Supported Discharge and home rehabilitation after stroke. Four main categories (on the left side of the figure) and 11 subcategories (on the right side of the figure) described the patients’ expectations

The analysis resulted in four categories and 11 subcategories (see Fig. 1).

### Support towards independency

The participants expected the VESD team to support them in such a way that they would manage and feel comfortable being at home. The expectations of the rehabilitation varied and full recovery was an overall wish by the participants. All of the participants were represented in this category.

**Getting support to manage at home** was stated as a positive aspect of the visits of the VESD team. The participants expected the team to check their home environment and how they managed there.

“*The first few days after I get home, they should be able to work out what I can’t manage to do, what I’m going to need help with*”. (Participant 4).

Another common expectation of the team was to support the participants in their daily activities.

*“…we decided that on the first day that I was going to cook and she was going to be with me. How to get it to work and the like. // Make something myself, lunch or something. I’m going to try to do it myself, but they would be, she would be with me.”* (Participant 7).

Some participants expected the VESD team to prescribe assistive devices to facilitate their everyday life as well as to put them in contact with people who provide the assistive devices. The VESD team was also expected to support the participants to master the environment.

*“… if it (the rehabilitation) happens at home, they can see what my place looks like. So they can adapt (my rehab) to it (the home environment) and make sure that I can master my environment more easily than I could do here (in hospital).”* (Participant 2).

Another aspect mentioned was that the visits from the VESD team were expected to contribute to a feeling of being secure at home.

*“… it feels secure to know that they are coming home, that they, I know that on Tuesday that she’s coming at 10 am, or whatever time it is (appointed time). So I know that they are coming here (to my home).”* (Participant 3).

**To get support to regain one’s former abilities** was described as an important aspect with regards to the rehabilitation provided by the VESD team. For some participants, the most important goal was to regain muscular strength and control.

*“… the most important is to get strength in my left arm and be more steady on my foot.”* (Participant 6).

For others, their rehabilitation goals also involved aspects of cognitive function as well as activity and participation in their lives.

*“… what I wanted was to rebuild my physical and mental strength so I get back to my regular activities and my regular life.”* (Participant 10)*.*

Yet another wish that was mentioned was to be supported to recover fully from their stroke.

*“I’m hoping and hoping and hoping, because I don’t know yet, I hope that I, that I can be completely recovered, as before.”* (Participant 7).

Functional training and self-exercise program provided by the VESD team were other kinds of support expected as means to regain one’s former abilities. The professional competence of the VESD team was stated as important for the rehabilitation outcome. The team was expected to be experienced and competent in stroke rehabilitation and this was highly appreciated among the participants.

*“It is sensible that people are coming who now what they are doing with this kind of stuff.”* (Participant 8).

### A new and unknown home situation

Six of the participants described expectations of coming home to a new and unknown situation even though they were returning to their homes. This was due to their changed abilities and to the different environmental conditions at home compared to at the hospital environment.

**Having changed capacities** compared to before the stroke, was expected to lead to an unfamiliar situation at home.

*“… I’ve always been independent // so it is a completely new situation and I can’t know how it is going to be // it might be great or it can go badly.”* (Participant 6).

For some participants, this led to insecurity about managing in their new home situation. Another consequence of the changed capacities was a fear of falling, which they expected would negatively affect their ability to manage at home and, would lead to activity limitations.

**Different environmental conditions** at home versus at hospital, was another reason participants felt they were at the beginning of something new and unknown. Some examples of these different conditions were the adapted environment for patients with stroke in hospital compared to at home and not having access to help from hospital staff 24 h a day when they were at home. As a result, some participants felt insecure about their ability to manage to perform the necessary activities of daily living at home.

*“… I’m worried about going to the toilet, // the worry of waking in the middle of the night and needing to go to the toilet. (At home) I’m the only one who can help me.”* (Participant 4).

### Returning to one’s setting

Eight of the participants talked about longing to go home, where they would become autonomous and capable people again.

**Being in a familiar environment** was described as desirable. The participants longed to be in their own surroundings, to have access to their own things, and to see their family again.

*“… living in my own house and doing my own stuff and being able to communicate with the rest of the world, because I don’t have any internet access here”.* (Participant10).

**Regaining one’s personal autonomy** was also a positive aspect of coming home. The participants looked forward to being the one deciding their daily routines again, and restarting their old habits that they could not purse while in hospital.

*“… being able to do what I want, like, I haven’t been able to open the window like I would normally do in the morning and those kind of things.”* (Participant 3).

**Being capable** was another aspect mentioned with regards to returning to one’s own setting. This was described by nearly all of the participants, in stark contrast to the feelings of insecurity that they expressed about being able to manage at home. More than half of the participants stated that they expected to be able manage the necessary activities of daily living at home.

*“I’m certain that I will be able (to go out by myself) when I get home, I can do it”* (Participant 8).

Being at home was even expected to facilitate managing daily activities and returning to everyday life.

*“So at home, you know where things are, here (in hospital) you need to go and look for… // If you need anything you have to, in other words they don’t have any particular spot. // It’s a bit easier at home.”* (Participant 9).

Another aspect dealt with capability with regards to issues of responsibility for and continuing with rehabilitation exercises.

*“… so you are forced to do more of your rehab exercises on your own (at home). Here (in hospital) you have an appointment, they’re coming, so we’re doing this now and doing that now. When I’m on my own (at home), that’s when it counts, and I know, I’m quite energetic so I think it’s going to go really well (with the training at home).”* (Participant 1).

### A new everyday life

Nine of the participants expressed that they wanted to resume their everyday life when they came home. Though, some expected to be dependent on others, which raised mixed feelings. Others highlighted the interference in everyday life by family members or the VESD team. Resuming their everyday life was expected to be challenging due to the consequences of the stroke on their functional abilities and emotional security. Different strategies to deal with these difficulties were described.

**Starting the “old” life again** when coming home was considered as important by almost all participants. They hoped to be able to perform the same activities and to participate in life just like they had done before the stroke.

*” I usually go to the tobacconist and bets on horses and I expect that I’ll continue doing that. It’s just a small hill to get up and shouldn’t be too hard for me. What’s going to happen is that you‘ll get back to what you were doing before.”* (Participant 8).

**Being dependent on others** was another main issue brought up by the participants. Some expected to be reliant on relatives to take care of the daily tasks.

*“… the computer, paying bills, my son made sure I did it (made sure the numbers were entered correctly), my son helps me.”* (Particpant 5).

Relatives and neighbors were also expected to be important for the participants feeling emotionally secure at home.

*“… I’ve got a phone so I can keep in contact with the outside world. And the neighbors, I keep in contact with them as well, if anything happens. So that’s also a bit of comfort. And my daughter’s there now too, so that’s also a comfort”.* (Participant 6).

Other expectations concerned assistance from the local community health service, which was considered important for managing to perform the necessary activities at home.

*“… if someone (from the community service) can go shopping, maybe you can cook by yourself. // If they shop for you, which I hope they do, because in the first few days (at home), I’m not sure I can do it by myself”.* (Participant 8).

Another way participants expected to be able to resume everyday life was by using the transportation service provided from the local community. They had mixed feelings with both worries and confidence described, that they would get the help they wanted from the community.

**Interference in the personal autonomy** was another aspect mentioned with regards to their new day-to-day life. There was an expectation that their family members could influence their behavior at home.

*“Yeah, I’ve got the kids stopping me now, // so you get reminded a fair bit. I’ve already been (reminded), they say that ‘you should think about yourself’, there’s lots of that. // So you have to take it a bit easy. // They are firm (with me), they’re going to be (firm with me)… // I am going to listen to them, // I have to, // it isn’t great (to be told what to do by your kids). I’m not hurt by it, but, you know, when you aren’t used to it.”* (Participant 1).

Another thing that the participants expected was that the VESD team would interfere, implying that team visits would intrude on their daily habits.

*“… when I’m free (not working) I usually go and have a lie down after I’ve eaten, in the middle of the day. // But of course there are a couple of visits planned with them (the team) at 1 pm next week.”* (Participant 3).

**Dealing with one’s new condition** referred to participants’ expectations that they will face challenges in their new everyday life due to the stroke’s consequences on their functional abilities and emotional security.

Some of these expectations concerned that their reduced functional abilities would lead to activity limitations and participation restrictions at home, compared to how they remembered their life prior the stroke, this gave raise to worries and frustrations.

*“Yes, it’s a bit like, you can’t just go out and shop (for groceries), I can’t just sit in the car when I want to, it will make me angry. Yeah, that I can’t do it, I don’t get sad, or depressed, it’s not that, I will just be angry I imagine. Yeah, and this leg here, this damned leg, // … when you are used to being able to manage and do everything by yourself…”* (Participant 1).

Other expectations were around the challenges related to their emotions about the stroke event, like fear of a recurrent stroke. These emotional challenges were also expected to result in activity limitations and restricted participation.

*“It’s just that, going out of your apartment, now I don’t want to do that. I mean if I fell over there, maybe no-one will notice and realize ‘she’s unwell’ //, or ring for an ambulance, so I’m a bit scared of that…”* (Participant 3).

Several different strategies were described to deal with one’s new conditions. Many of the participants wanted to take one day, and one thing at a time, and have a slow return to their usual life. So one strategy mentioned was for activities at home, they planned to postpone or change the way they did them.

*“Maybe I won’t be able to peel potatoes and cut meat the way I’m used to, but that can wait and I’ll buy something that’s ready-made, it’ll be fine.”* (Participant 8).

Another tactic they described was to perform activities more slowly and with more control than before or to do one thing at a time. This was expected to be a difficult strategy to learn, but was considered as important.

## Discussion

Our findings were that the participants had confidence that the VESD team would support them in gaining independence. Mixed expectations for coming home were described, which revealed ambivalence about being discharged home from hospital.

The expectations that the participants described about the intervention showed that they had confidence that the support from the VESD team would help them to be able to manage different activities, both in- and outside their home, and that this support would help enable their functional recovery. Similar findings have been described in a previous study of ESD [16]. The health professionals at the hospital might build on this expected trust, in order to reduce the patients’ insecurity not to manage at home and, thereby facilitating their transition home.

Also highlighted in the current study, was the expectation that the visits from the VESD team would reduce the participant’s insecurity. Previous research has shown that anxiety occurs frequently after stroke [26], and that this can potentially affect the rehabilitation process. Therefore, home rehabilitation from a hospital based team could be beneficial in multiple ways such as mastering the home environment, reducing emotional distress and thereby improving the rehabilitation outcome.

Previous studies have shown that, according to the patients, rehabilitation after stroke mainly deals with physical disabilities and less with cognitive impairments [27–29]. Training of cognitive functions such as memory has been identified as an area of potential improvement by patients receiving an ESD and home-based rehabilitation [17]. In the present study, there were few expectations expressed by the participants regarding support to deal with cognitive impairments. This was somewhat surprising, as half of the participants scored between 15 and 23 points on the MoCA, since early cognitive impairments are found to be a predictor for long-term depression and reduced life quality after stroke [30] and one of the areas that should be addressed in rehabilitation after stroke, according to national guidelines in Sweden [13].

Mixed expectations were described about coming home very early from hospital after a stroke, which supports the findings from earlier studies [7, 31]. In the present study, some of these expectations were contradictory, concerning foremost the idea of one’s home as an unknown place raising feelings of insecurity and fear, in contrast to the home as a familiar setting assumed to facilitate recovery and the rehabilitation process. The expectation of being discharged to something unknown has been described in an earlier study in persons with stroke [31], because the old habits and routines would no longer apply in the situation. From an International Classification of Functioning, Disability and Health (ICF) perspective [32], this new and unknown home situation can be explained by the changed interaction between body functions, activities and participation, as well as between these domains and environmental factors. This change in environmental conditions when being discharged home, challenges the functional abilities of the patients and has been described as a dividing line between a known and an unknown situation [31].

As stated above, the participants sometimes described mixed feelings about coming home, insecurity and fear as well as longing. These findings are in contrast to previous studies of patients’ experiences of ESD [16, 17], describing only positive feelings for coming home early after stroke. However, the prospective design of the current study in contrast to the retrospective design in the earlier studies [16, 17], could contribute to these differences. Being afraid of a new life situation could be seen as normal reaction. Recognizing and normalizing these feelings might therefore facilitate the process of coming home and make the first period less uncertain.

The positive expectations towards coming home described in the current study, such as being a capable and autonomous person, being in a familiar environment and taking up one’s old everyday life, has also been described in earlier studies [7, 31]. Patients have identified the transition home from hospital after a stroke as an important milestone in the rehabilitation process and as an important step for further recovery and rehabilitation [7]. However, unrealistically high expectations of recovery and rehabilitation during the first weeks after stroke have been described in earlier studies [31, 33]. In the present study, such unrealistically high expectations might be uncovered in the subcategory “to be capable”. As patients were confident that they would manage ADL as soon they came home, even though they were not able to perform these activities in hospital. Such unrealistic expectations may lead to the return home becoming a disappointment and the participants might lose motivation for further training. Unrealistically high expectations might be due to impaired cognitive functions as a consequence of the stroke. It may therefore be important for both patients and health professionals to discover any reduced self-introspection capabilities and unrealistically high expectations of recovery, as well as encouraging more realistic expectations, in order to improve the rehabilitation process [34]. On the other hand, hope has been identified as an important factor in the recovery after stroke [35]. To be open-minded with regards to seemingly unrealistic expectations and goals might, therefore, have a positive impact on the recovery.

Due to logistical reasons, two patients had not had their planning meeting before the interview for the current study. Accordingly, their rehabilitation goals had not been formulated and agreed upon. This might have affected their expectations about the content of the home rehabilitation intervention. Therefore, they may have described somewhat different experiences if the interviews had occurred after their planning meeting.

The interviews were on average 30 min, ranging from 9 to 53 min. Three of them were significantly shorter than the others (<15 min). These three interviews were conducted according to the protocol and the most vital information for the study aim was given by the participants. The participants did not expand on their responses, which may be explained by cognitive impairments. However, patients with mild cognitive impairments are common at the acute stroke ward, and were therefore included in the study in order to resemble the clinical setting.

The participants were of both sexes and both persons living alone and sharing households were included. They had a variety of cognitive impairments as seen in the MoCA scores ranging from 15 to 27, and scored across the whole range of the BI scale (50–100), thus showing different levels of functional disabilities. The participants’ diversity with regards to impairment levels, activity limitations as well as environmental and personal factors, may have contributed to an enrichment of the result. All participants were older than 62 years and most of them were born in Sweden, so it is reasonable to assume that different expectations may have been described by younger persons with stroke or from persons born abroad with a different cultural background.

A sample size of 10 participants can be considered as small. However, the intention of qualitative research is not to generalize and there is no commonly accepted sample size but other criteria are of importance, such as richness and variation in data [36]. When studying a restricted population, such as the intervention group of 120 patients in GOTVED, a lower number of participants may be needed [37]. In this case, we considered that the variation with regards to personal characteristics, impairments, activity limitations as well as the information richness and variation in data, were appropriate and sufficient to answer the aim of this study. Also, many patients in this situation are affected by fatigue and emotional distress. Therefore, we found it unethical to interview more patients than necessary, especially since they were exposed to several additional tests and questionnaires in front of discharge.

A strength of the current study was the inclusion of persons with language and speech disorders. This group of patients is often excluded from interview studies for practical reason, even though these deficits are common after stroke, and are often seen in clinical settings. Finally, the patients’ descriptions are based on experiences of the Swedish health care system, which must be regarded when considering transferability into other cultural and societal contexts.

## Conclusions

This study showed that the thought of coming home gave rise to mixed expectations such as feeling insecure about their ability to manage at home and fear for a recurrent stroke and, on the other hand, longing to one’s own setting. Moreover, the participants had a high degree of confidence in the VESD team with regards to the support they expected to receive at home. To facilitate discharge, the health professionals at the hospital may build on this expected trust, in order to reduce the patients’ feelings of insecurity for coming home. In addition, exploring the patients’ expectations thoroughly in front of discharge might be beneficial, as certain feelings and thoughts could complicate or support the home coming process. Thus, a greater attention on such expectations might facilitate the patient’s transition from hospital to home after stroke.
